# Assessing the roles of shape prototypicality and sexual dimorphism in ratings of the trustworthiness of faces

**DOI:** 10.1038/s41598-023-42990-6

**Published:** 2023-09-20

**Authors:** Kathlyne Leger, Junzhi Dong, Lisa M. DeBruine, Benedict C. Jones, Victor K. M. Shiramizu

**Affiliations:** 1 School of Psychological Sciences and Health, University of Strathclyde, Glasgow, UK; 2 School of Psychology and Neuroscience, University of Glasgow, Glasgow, UK

**Keywords:** Psychology, Human behaviour

## Abstract

Perceptions of the trustworthiness of faces predict important social outcomes, including economic exchange and criminal sentencing decisions. However, the specific facial characteristics that drive trustworthiness perceptions remain poorly understood. Here we investigated this issue by exploring possible relationships between ratings of the trustworthiness of face images and objective assessments of two aspects of face shape that researchers have previously argued are important for perceptions of trustworthiness: distinctiveness and sexual dimorphism. Here we report that faces with more distinctive shapes are rated as less trustworthy, but that sexual dimorphism of face shape is not significantly correlated with trustworthiness ratings. These results suggest that distinctiveness of face shape plays a more important role in trustworthiness perceptions than does sexual dimorphism and suggest that perceptions of trustworthiness may stem, at least in part, from the ‘anomalous-is-bad’ stereotype.

## Introduction

Perceptions of trustworthiness based on facial appearance predict important social outcomes. For example, in an economic game assessing trusting behaviour, people were more likely to trust individuals whose faces were judged more trustworthy by third-party raters^1^. Moreover, convicted murderers in a US state with the death penalty (Florida) were more likely to receive death sentences if their faces were judged less trustworthy by third-party raters^2^. Despite this evidence that trustworthiness ratings of faces predict social outcomes, the specific physical characteristics that predict perceptions of trustworthiness are unclear.

Sexually dimorphic shape information is one facial characteristic that researchers have suggested may play an important role in perceptions of trustworthiness. Several studies have reported that versions of face images in which shape characteristics had been feminised using computer-graphic methods (i.e., versions in which female sex-typical shape characteristics had been increased) were perceived to be more trustworthy than masculinised versions [e.g.,^3–5^; but see also^6^]. However, this method for investigating possible links between physical characteristics and social judgments of faces has recently been criticised^7,8^. Indeed, studies of attractiveness and dominance judgments have shown that, although studies using experimentally manipulated face images often show large effects of shape manipulations on perceptions, effects are considerably smaller (and often not significant) when natural (i.e., unmanipulated) face images are rated and the shape characteristics being investigated are objectively assessed from the images^7–10^. Other work has suggested that the forced-choice method assesses perceptual-discrimination and/or face-matching ability, rather than social judgments, per se^11,12^. These patterns of results suggest that findings for perceptions of face stimuli manipulated on a single dimension do not necessarily generalise to ratings of natural face images^7–13^. Furthermore, although some studies have reported that trustworthiness ratings of face images are positively correlated with ratings of their femininity and negatively correlated with ratings of their masculinity [e.g.,^14^], other studies suggest that masculinity and femininity ratings of faces are influenced by characteristics that are not sexually dimorphic^9,10,15^. For these reasons, we suggest that the role that sexually dimorphic shape information plays in perceptions of the trustworthiness of face images is currently unclear.

An alternative, but not necessarily mutually exclusive, explanation for perceptions of trustworthiness stems from the ‘anomalous-is-bad’ stereotype^16^. This stereotype refers to the tendency for perceivers to erroneously ascribe negative personality traits to individuals with atypical physical appearances^16^. Although tests for the existence of such a stereotype have generally focused on the effects of prominent facial anomalies^16–18^, it is possible that this stereotype also extends to the effects of more subtle deviations from prototypical face shapes on perceptions of trustworthiness. Consistent with this possibility, Ryali et al.^19^ recently reported that the statistical typicality of face images was positively correlated with ratings of their trustworthiness [see also^20^.

In light of the above, we investigated possible relationships between ratings of the trustworthiness of natural (i.e., unmanipulated) face images and objective assessments of both the atypicality (i.e., distinctiveness) and sexual dimorphism of their face shapes. Trustworthiness ratings and face stimuli were taken from an open-access face-image database^21^ and sexual dimorphism and distinctiveness of face shape were assessed using image-analysis methods widely used in face-perception research e.g.,^22–24^].

## Results

All analyses were carried out using R^25^, with the packages kableExtra 1.3.4^26^, lme4^27^, lmerTest 3.1–3^28^, jtools 2.2.3^29^, psych 2.2.5^30^, robustHD 0.7.3^31^, and tidyverse 1.3.1^32^. All data, full outputs, and analysis code are publicly available on the Open Science Framework (https://osf.io/htbjv/).

We tested for possible relationships between trustworthiness ratings and both sexual dimorphism and distinctiveness scores using a linear mixed effects model. Trustworthiness ratings served as the dependent variable. The model included main effects of sexual dimorphism scores, distinctiveness scores, rater sex (effect coded so that 0.5 corresponded to male raters and −0.5 corresponded to female raters), and face sex (effect coded so that 0.5 corresponded to male faces and −0.5 corresponded to female faces) as predictors, as well as all possible two- and three-way interactions, excluding those involving both of the continuous predictors (i.e. no interactions including both sexual dimorphism and distinctiveness scores were included in the model). The model also included, by-rater and by-stimulus random intercepts, by-rater random slopes for sexual dimorphism and distinctiveness (face sex varied between raters), and by-stimulus random slopes for rater sex. Sexual dimorphism and distinctiveness scores were standardised prior to analyses by converting them to z scores. Results are summarised in Table 1.

**Table 1:** Summary of results from our linear mixed effects model analysing trustworthiness ratings.

	Estimate	SE	t	df	p
Sexual dimorphism	0.018	0.052	0.343	100.435	0.732
Distinctiveness	−0.142	0.052	−2.732	101.134	0.007
Face sex	−0.007	0.128	−0.053	227.701	0.958
Rater sex	0.157	0.080	1.958	403.924	0.051
Sexual dimorphism x Rater sex	−0.002	0.021	−0.103	98.694	0.918
Sexual dimorphism x Face sex	0.145	0.104	1.395	100.435	0.166
Distinctiveness x Rater sex	0.014	0.022	0.616	112.017	0.539
Distinctiveness x Face sex	−0.100	0.104	−0.959	101.134	0.340
Face sex x Rater sex	0.298	0.160	1.858	403.924	0.064
Sexual dimorphism x Rater sex x Face sex	0.011	0.042	0.252	98.694	0.801
Distinctiveness x Rater sex x Face sex	−0.035	0.044	−0.788	112.017	0.432

**Figure 1:**
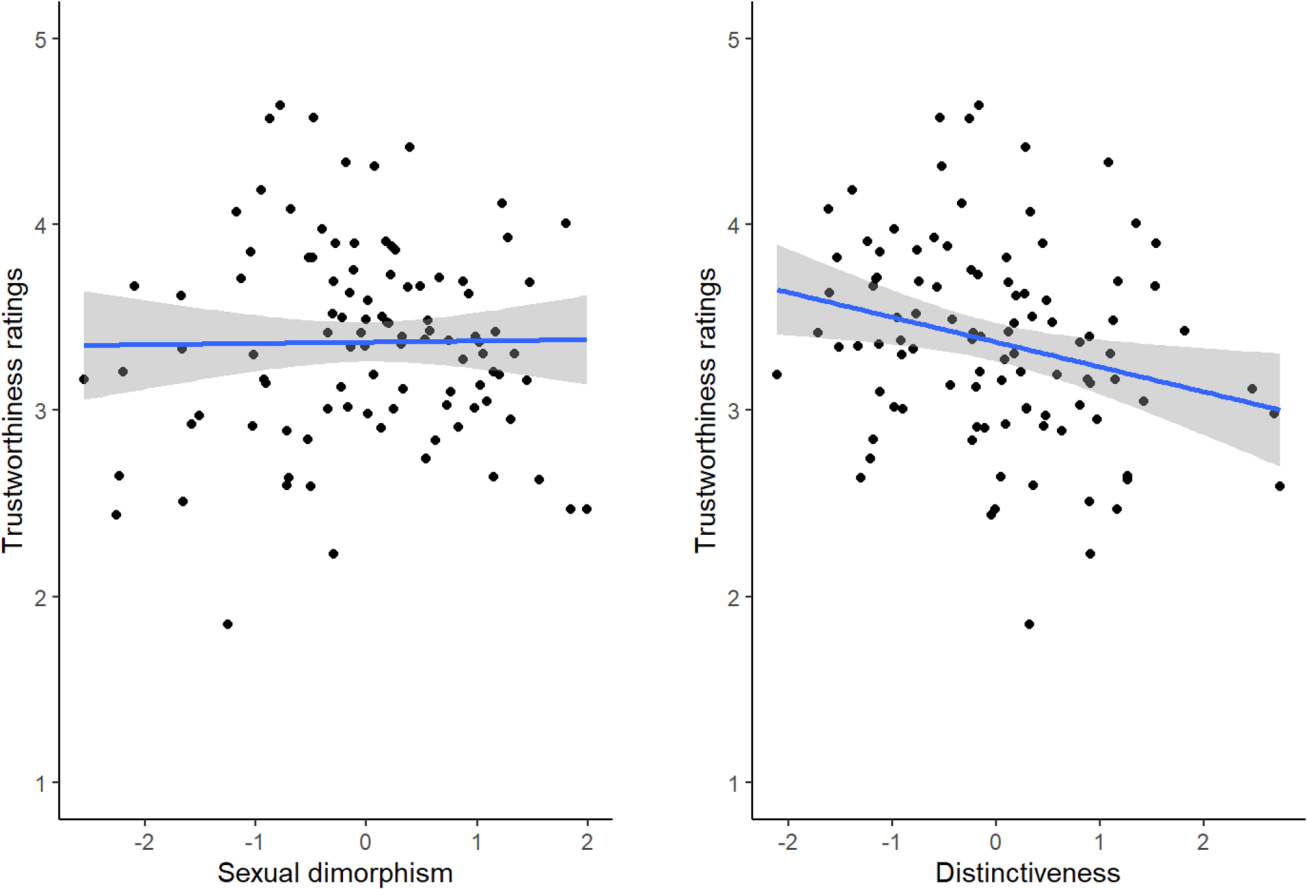
The non-significant relationship between sexual dimorphism and trustworthiness ratings (left) and the significant relationship between distinctiveness and trustworthiness ratings (right). The shaded areas show 95% confidence intervals.

Our analysis revealed a significant negative main effect of distinctiveness, indicating that faces with less distinctive face shapes were rated more trustworthy. By contrast, the main effect of sexual dimorphism was not significant. Neither the main effects of rater sex or face sex, nor any of the two- or three-way interactions were significant. Figure 1 shows the relationships between trustworthiness ratings and both sexual dimorphism and distinctiveness.

Additional analyses in which the same model was run including either (1) all possible main effects and interactions or (2) only the main effects of distinctiveness and sexual dimorphism as predictors showed the same pattern of results as our main analyses. In both cases, the only significant effect was the negative effect of distinctiveness. Full results for both of these analyses are available at https://osf.io/htbjv/.

## Discussion

We investigated possible relationships between ratings of the trustworthiness of natural (i.e., unmanipulated) face images and objective assessments of both the distinctiveness (i.e., atypicality) and sexual dimorphism of their face shapes. There was a significant negative relationship between distinctiveness of face shape and trustworthiness (see Fig. 1), indicating that more trustworthy-looking faces had less distinctive (i.e., more prototypical) face shapes (see Fig. 1). By contrast, the relationship between sexual dimorphism of face shape and trustworthiness ratings was not significant (see Fig. 1).

The significant negative relationship between trustworthiness ratings and shape distinctiveness is consistent with previous research reporting that trustworthy-looking faces tend be more typical^19,20^. It is also consistent with the ‘anomalous-is-bad’ stereotype^16^, which refers to the tendency for perceivers to ascribe negative personality traits to individuals with atypical physical appearances^16^. While most previous research on the ‘anomalous-is-bad’ stereotype has examined the effects of prominent anomalies [e.g.,^16–18^], our results suggest that this stereotype also extends to the effects of more subtle deviations from prototypical face shapes. More fundamentally, our results suggest that distinctiveness of face shape contributes significantly to trustworthiness perceptions, even when stimuli are natural (i.e., unmanipulated) faces that vary simultaneously on other dimensions.

Our null result for sexual dimorphism of face shape and trustworthiness ratings contrasts with previous studies reporting that versions of face images in which female sex-typical shape characteristics had been increased were perceived as more trustworthy than versions of face images in which male sex-typical shape characteristics had been increased [e.g.,^3–5^, but see also^6^]. However, recent work on attractiveness and dominance judgments has found that the large effects typically observed when face-shape characteristics are manipulated in face images are not observed when individual, unmanipulated faces were rated and face-shape characteristics measured objectively from face images^7–10^. Our null result for trustworthiness ratings and sexual dimorphism suggests that the extent to which sexually dimorphic aspects of face-shape influence perceptions of trustworthiness is also determined by study design and that the large effects observed for manipulated shape characteristics do not generalise to ratings of natural (i.e., unmanipulated) face images. Perhaps more importantly, our null result for sexual dimorphism does not support the proposal that sexually dimorphic face-shape is an important cue for perceptions of trustworthiness.

Many previous studies have compared the effects of manipulated shape characteristics on social judgments of faces across world regions. For example, Perrett et al.^4^ found that experimentally manipulating sexually dimorphic shape information in face images had similar effects on social judgments of faces made by UK and Japanese participants (e.g., feminising shape characteristics made faces appear more trustworthy to both UK and Japanese participants). By contrast, using similar methods, Scott et al.^33^ found that the size and even direction of the effects of sexual dimorphism on social judgments of faces could differ markedly across world regions, with some regions showing the opposite pattern of results to those observed in western regions. Results from studies investigating effects of other face-shape characteristics (e.g., distinctiveness) in different cultures are similarly mixed [reviewed in^34^]. A limitation of these studies is that they typically employed experimentally manipulated face images as stimuli and assessed perceptions using forced-choice methods. Establishing whether the relationships between ratings of individual faces and objectively measured face-shape parameters are similar or different across world regions may provide a clearer picture of cultural differences and similarities in the cues that drive social judgments of faces. Similarly, while both the stimuli and raters in our study reflected a relatively narrow range of ages, future work with stimuli and raters that are more diverse in terms of their age would allow researchers to test for possible age-related differences in the cues that drive social judgments of faces. Indeed, recent work suggests that trustworthiness ratings of faces are influenced by the interaction between stimulus and perceiver ages^35^.

A further open question is the extent to which our results for perceptions of trustworthiness are specific to trustworthiness ratings or generalise to ratings of other prosocial traits. Previous studies have reported that subjecting ratings of faces on a wide range of traits (e.g., aggressiveness, attractiveness, caringness, confidence, dominance, emotional stability, unhappiness, intelligence, meanness, responsibility, sociability, trustworthiness, weirdness) to Principal Component Analysis (PCA) reveals that social judgments of faces are underpinned by two core perceptual dimensions [e.g.,^36,37^]. The first of these dimensions, often labelled Valence, is highly correlated with ratings of traits such as trustworthiness and attractiveness and is thought to reflect impressions of individuals’ motivations to inflict harm on others [e.g.,^36,37^]. The second of these dimensions, often labelled Dominance, is highly correlated with ratings of dominance and aggressiveness and is thought to reflect impressions of individuals’ capacities to inflict harm on others [e.g.,^36,37^]. Given the intercorrelated nature of many trait ratings, future work investigating predictors of these core perceptual dimensions, rather than individual traits, may provide particularly useful insight into the factors that drive social judgments of faces.

To summarize, we investigated possible relationships between ratings of the trustworthiness of natural face images and objective assessments of the distinctiveness and sexual dimorphism of their face shapes. By contrast with previous research that used experimentally manipulated face stimuli [e.g.,^3–5^], we found no evidence that sexual dimorphism of face shape plays an important role in perceptions of trustworthiness. However, our results suggest that distinctiveness of face shape contributes significantly to perceptions of trustworthiness and appears to play a more important role in trustworthiness perceptions than does sexual dimorphism. Collectively, these results underline the importance of establishing whether findings for experimentally manipulated face stimuli generalise to judgments of natural (i.e., unmanipulated) face images.

## Methods

### Ethics

All procedures were approved by the School of Psychological Sciences and Health (University of Strathclyde) Ethics Commitee, all work was undertaken in accordance with the Declaration of Helsinki, and all participants provided informed consent.

### Trustworthiness ratings

Ratings of trustworthiness were taken from an open-access face-image database^21^. Two hundred men and 200 women (mean age = 25.11 years, SD = 6.00 years) were randomly allocated to rate either 50 male face images or 50 female face images for trustworthiness using a 1 (much less trustworthy than average) to 7 (much more trustworthy than average) scale. Trial order was fully randomised. Images had been standardised on pupil position and clothing masked prior to rating and the individuals photographed posed with neutral expressions, front-on to the camera, and with direct gaze. Male and female face images depicted young adult white men (mean age = 24.2 years, SD = 3.99 years) and young adult white women (mean age = 24.3 years, SD = 4.01 years), respectively. Ratings were collected using the Experimentum data-collection platform^38^. Cronbach’s alphas were high for ratings of both male faces (Cronbach’s alpha = 0.97) and female faces (Cronbach’s alpha = 0.98). Cronbach’s alphas were also high when calculated separately for ratings of male faces by male raters only (Cronbach’s alpha = 0.96), ratings of male faces by female raters only (Cronbach’s alpha = 0.96), ratings of female faces by male raters only (Cronbach’s alpha = 0.93), and ratings of female faces by female raters only (Cronbach’s alpha = 0.95).

### Sexual dimorphism of face shape

Sexual dimorphism of face shape was objectively measured for each of the 50 male and 50 female face images using the facefuns package^39^ in R^25^. This method that has been used in many previous studies to assess sexual dimorphism of face shape [e.g., ^9,15,22–24^]. Shape components were first derived from Principal Component Analysis (PCA) of 132 Procrustes-aligned landmark points (see Holzleitner et al.^23^ for a diagram showing these facial landmarks) on each of the 50 male and 50 female face images. Scores representing sexual dimorphism of face shape were then calculated from each photograph using a vector analysis method [e.g.,^9,15,22–24^]. This method uses the shape principal components to locate each face on a female-male continuum. The female-male continuum was defined by calculating the average shape information of the 50 female faces and the average shape information of the 50 male faces. Sexual dimorphism scores were then derived by projecting each image onto this female-male vector. Higher scores indicated more masculine face shapes.

### Distinctiveness of face shape

Distinctiveness scores were also calculated from each photograph using the facefuns package^39^ in R^25^. This technique has been used to measure face-shape distinctiveness in many previous studies [e.g.,^9,22,23,40^]. This method uses the shape principal components described in the previous section of our methods to measure the distance each face lies from the mathematical average shape for the sample of faces of the same sex. That is, the average shape values for the same-sex sample were calculated and, for each image, the Euclidean distance from the average was derived. Higher scores indicate that the face lies a further distance away from the average (i.e., had a more distinctive shape). We measured distinctiveness scores for male and female faces separately in light of evidence that faces are primarily processed relative to sex-specific prototypes [e.g.,^41,42^].

### Correlation between sexual dimorphism and distinctiveness scores

Analyses using Pearson correlations indicated that sexual dimorphism and distinctiveness scores were not significantly correlated for the whole sample of 100 faces (r = 0.03, N = 100, *p* = 0.70), the sample of 50 male faces (r = 0.15, N = 50, *p* = 0.31), or the sample of 50 female faces (r =−0.07, N = 50, *p* = 0.63).

## Data Availability

Data and stimuli are publicly available on the Open Science Framework at https://osf.io/htbjv/ and https://osf.io/a3947/, respectively.

## Code availability

Analysis code is publicly available on the Open Science Framework at https://osf.io/htbjv/.

## References

[1] Van’t Wout M, Sanfey AG (2008). Friend or foe: the effect of implicit trustworthiness judgments in social decision-making. Cognition.

[2] Wilson JP, Rule NO (2015). Facial trustworthiness predicts extreme criminal-sentencing outcomes. Psychol. Sci..

[3] Buckingham G, DeBruine LM, Little AC, Welling LL, Conway CA, Tiddeman BP, Jones BC (2006). Visual adaptation to masculine and feminine faces influences generalized preferences and perceptions of trustworthiness. Evol. Hum. Behav..

[4] Perrett DI, Lee KJ, Penton-Voak I, Rowland D, Yoshikawa S, Burt DM, Akamatsu S (1998). Effects of sexual dimorphism on facial attractiveness. Nature.

[5] Smith FG, Jones BC, Little AC, DeBruine LM, Welling LL, Vukovic J, Conway CA (2009). Hormonal contraceptive use and perceptions of trust modulate the effect of relationship context on women’s preferences for sexual dimorphism in male face shape. J. Evol. Psychol..

[6] Alharbi SA, Holzleitner IJ, Lee AJ, Saribay SA, Jones BC (2020). Women’s preferences for sexual dimorphism in faces: data from a sample of Arab women. Evol. Psychol. Sci..

[7] Jones AL, Jaeger B (2019). Biological bases of beauty revisited: the effect of symmetry, averageness, and sexual dimorphism on female facial attractiveness. Symmetry.

[8] Lee AJ, De La Mare JK, Moore HR, Umeh PC (2021). Preference for facial symmetry depends on study design. Symmetry.

[9] Dong J, Leger K, Shiramizu VK, Marcinkowska UM, Lee AJ, Jones BC (2023). The importance of face-shape masculinity for perceptions of male dominance depends on study design. Sci. Rep..

[10] Scott IM, Pound N, Stephen ID, Clark AP, Penton-Voak IS (2010). Does masculinity matter? The contribution of masculine face shape to male attractiveness in humans. PLoS ONE.

[11] Lewis MB (2020). Challenges to both reliability and validity of masculinity-preference measures in menstrual-cycle-effects research. Cognition.

[12] Lewis MB (2017). Fertility affects asymmetry detection not symmetry preference in assessments of 3D facial attractiveness. Cognition.

[13] Jones, B. C., Jones, A., & Shiramizu, V. K. M. Mapping physical characteristics in face images to social judgments. British Journal of Psychology, in press. (2022). 10.1111/bjop.12617 36463493

[14] Hester N, Jones BC, Hehman E (2021). Perceived femininity and masculinity contribute independently to facial impressions. J. Exp. Psychol. Gen..

[15] Bartlome, R. I., & Lee, A. J. Facial attractiveness, but not facial masculinity, is used as a cue to paternal involvement in fathers. Adaptive Human Behavior and Physiology, 1–16. (2023). 10.1007/s40750-023-00217-y PMC10234791 37360188

[16] Workman CI, Humphries S, Hartung F, Aguirre GK, Kable JW, Chatterjee A (2021). Morality is in the eye of the beholder: the neurocognitive basis of the “anomalous-is-bad” stereotype. Ann. N. Y. Acad. Sci..

[17] Workman CI, Smith KM, Apicella CL, Chatterjee A (2022). Evidence against the “anomalous-is-bad” stereotype in Hadza hunter gatherers. Sci. Rep..

[18] Jamrozik A, Oraa Ali M, Sarwer DB, Chatterjee A (2019). More than skin deep: judgments of individuals with facial disfigurement. Psychol. Aesthet. Creat. Arts.

[19] Ryali CK, Goffin S, Winkielman P, Yu AJ (2020). From likely to likable: The role of statistical typicality in human social assessment of faces. Proc. Natl. Acad. Sci..

[20] Sofer C, Dotsch R, Wigboldus DH, Todorov A (2015). What is typical is good: The influence of face typicality on perceived trustworthiness. Psychol. Sci..

[21] DeBruine, L. M., & Jones, B. C. 3DSK face set with webmorph templates. Open Science Framework. (2022). 10.17605/OSF.IO/A3947

[22] Cai Z, Hahn AC, Zhang W, Holzleitner IJ, Lee AJ, DeBruine LM, Jones BC (2019). No evidence that facial attractiveness, femininity, averageness, or coloration are cues to susceptibility to infectious illnesses in a university sample of young adult women. Evol. Hum. Behav..

[23] Holzleitner IJ, Lee AJ, Hahn AC, Kandrik M, Bovet J, Renoult JP, Jones BC (2019). Comparing theory-driven and data-driven attractiveness models using images of real women’s faces. J. Exp. Psychol.: Human Percept. Perform..

[24] Komori M, Kawamura S, Ishihara S (2011). Multiple mechanisms in the perception of face gender: effect of sex-irrelevant features. J. Exp. Psychol. Hum. Percept. Perform..

[25] R Core Team R: A language and environment for statistical computing. R Foundation for Statistical Computing, Vienna, Austria. (2021). Retrieved from http://www.R-project.org/

[26] Zhu, H. kableExtra: Construct Complex Table with 'kable' and Pipe Syntax. (Version 1.3.4) [Computer software]. (2021). Retrieved from https://cran.r-project.org/web/packages/kableExtra

[27] Bates D, Maechler M, Bolker B, Walker S, Christensen RH, Singmann H, Dai B (2015). lme4: Linear mixed-effects models using Eigen and S4. R Package Version.

[28] Kuznetsova A, Brockhoff PB, Christensen RHB (2017). lmerTest package: tests in linear mixed effects models. J. Stat. Softw..

[29] Long, J. A. jtools: Analysis and Presentation of Social Scientific Data. (Version 2.2.0). [Computer software]. (2022). Retrieved from https://cran.r-project.org/web/packages/jtools

[30] Revelle, W. Psych: Procedures for personality and psychological research (Version 2.2.5). [Computer software]. (2022). Retrieved from https://CRAN.R-project.org/packagepsych

[31] Alfons, A. robustHD: Robust Methods for High-Dimensional Data (Version 0.7.3) [Computer software]. (2022). Retrieved from: https://cran.r-project.org/web/packages/robustHD/index.html

[32] Wickham, H. tidyverse: Easily Install and Load the 'Tidyverse' (Version 1.3.1). [Computer software]. (2021). Retrieved from https://cran.r-project.org/web/packages/tidyverse/index.html

[33] Scott IM, Clark AP, Josephson SC, Boyette AH, Cuthill IC, Fried RL, Penton-Voak IS (2014). Human preferences for sexually dimorphic faces may be evolutionarily novel. Proc. Natl. Acad. Sci..

[34] Apicella CL, Barrett HC (2016). Cross-cultural evolutionary psychology. Curr. Opin. Psychol..

[35] Pehlivanoglu D, Lin T, Lighthall NR, Heemskerk A, Harber A, Wilson RC, Ebner NC (2023). Facial trustworthiness perception across the adult life span. J. Gerontol.: Ser. B.

[36] Oosterhof NN, Todorov A (2008). The functional basis of face evaluation. Proc. Natl. Acad. Sci..

[37] Jones BC, DeBruine LM, Flake JK, Liuzza MT, Antfolk J, Arinze NC, Sirota M (2021). To which world regions does the valence–dominance model of social perception apply?. Nat. Human Behav..

[38] DeBruine, L. M., Lai, R., Jones, B. C., Abdullah, R. & Mahrholz, G. Experimentum (Version v.0.2). Zenodo. (2020). 10.5281/zenodo.2634355

[39] Hozleitner, I., J. & DeBruine, L. M. facefuns (Version 0.0.0.900) [Computer software]. (2021). Retrieved from https://iholzleitner.github.io/facefuns/index.html

[40] Lee AJ, Mitchem DG, Wright MJ, Martin NG, Keller MC, Zietsch BP (2016). Facial averageness and genetic quality: Testing heritability, genetic correlation with attractiveness, and the paternal age effect. Evol. Hum. Behav..

[41] Little AC, DeBruine LM, Jones BC (2005). Sex-contingent face after-effects suggest distinct neural populations code male and female faces. Proc. R. Soc. B.

[42] Rhodes G, Jaquet E, Jeffery L, Evangelista E, Keane J, Calder AJ (2011). Sex-specific norms code face identity. J. Vis..

